# C-852-01. Topical Sodium Hypochlorite Solution vs. Mafenide Acetate: A Comparative Analysis of Antimicrobial Effectiveness and Postoperative Burn Healing Outcomes

**DOI:** 10.1093/jbcr/irag033.061

**Published:** 2026-04-06

**Authors:** Mare G Kaulakis, Viktoria Voragen, Mia J Carrarini, Christopher J Fedor, Omar Zein, Sarah M Tepe, Simon Rodriguez, Mahi Pachgade, Francesco M Egro

**Affiliations:** University of Pittsburg Medical Center Mercy Burn Center, Pittsburgh, PA; University of Pittsburgh School of Medicine, Pittsburgh, PA; University of Pittsburgh Medical Center, Pittsburgh, PA; University of Pittsburg Medical Center Department of Plastic Surgery, Pittsburgh, PA; University of Pittsburg Medical Center Mercy Burn Center, Pittsburgh, PA; University of Pittsburgh Medical Center, Pittsburgh, PA; University of Pittsburgh School of Medicine, Pittsburgh, PA; Drexel University College of Medicine, Philadelphia, PA; University of Pittsburgh Medical Center, Pittsburgh, PA

## Abstract

**Introduction:**

Third-degree burns are especially prone to infection, often requiring topical antimicrobials like mafenide acetate and topical sodium hypochlorite solution. Despite promising results in models, direct comparisons in patients are lacking. This study aims to evaluate infection and mortality outcomes to address this gap.

**Methods:**

A retrospective analysis of patients over the age of 18 that were diagnosed with 3rd-degree burns at a single ABA verified burn center (2012-2025). Variables collected include demographic information, comorbidities, infection data, and incidence of complications and mortality. T-tests, Chi-squared tests, and regressions adjusting for age, TBSA, comorbidities, and other significant predictors were done.

**Results:**

A total of 292 patients were included (69.5% male, mean age 50.3 ± 17.2 years). There were no significant differences between the topical sodium hypochlorite group (n = 47) and the mafenide acetate group (n = 245) in age, sex, BMI, TBSA, or infection rates (all *p*>.05).

However, patients treated with topical sodium hypochlorite had significantly higher rates of wound healing complications (17.0% vs. 5.7%, *p*=.007), donor site complications (18.6% vs. 2.1%, *p*<.001), and partial graft loss (16.3% vs. 4.1%, *p*=.002).

In multivariable linear regression, topical sodium hypochlorite use was independently associated with a 0.96 increase in the number of surgeries (β = 0.96, 95% CI: 0.51–1.41, *p*<.001) and 6.07 more hospital days (β = 6.07, 95% CI: 1.94–10.21, *p*=.004) compared to mafenide acetate. No significant association was observed between treatment type and mortality (OR = 0.66, 95% CI: 0.10–4.25, *p*=.659).

**Conclusions:**

In this cohort, treatment with topical sodium hypochlorite solution was associated with increased surgical interventions, longer hospital stays, and higher rates of wound healing and donor site complications compared to mafenide acetate. However, there were no significant differences in mortality or infection rates between the two groups. These findings suggest that mafenide acetate may offer advantages in reducing postoperative complications and resource utilization for burn patients.

**Applicability of Research to Practice:**

These results highlight the impact of antimicrobial choice on patient outcomes and resource utilization in burn care.

**Funding for the study:**

N/A.

